# Beneficial Effects of *Bauhinia rufa* Leaves on Oxidative Stress, Prevention, and Treatment of Obesity in High-Fat Diet-Fed C57BL/6 Mice

**DOI:** 10.1155/2022/8790810

**Published:** 2022-11-25

**Authors:** Paola dos Santos da Rocha, Sarah Lam Orué, Daniel Ferreira Leite, Priscilla Pereira de Toledo Espindola, Nadla Soares Cassemiro, Denise Brentan da Silva, Carlos Alexandre Carollo, Valéria Nunes-Souza, Luiza Antas Rabelo, Jaqueline Ferreira Campos, Letícia Miranda Fernandes Estevinho, Edson Lucas dos Santos, Kely de Picoli Souza

**Affiliations:** ^1^Research Group on Biotechnology and Bioprospecting Applied to Metabolism (GEBBAM), Federal University of Grande Dourados, Rodovia Dourados-Itahum, Km 12, Dourados, MS 79804-970, Brazil; ^2^Laboratory of Natural Products and Mass Spectrometry, Federal University of Mato Grosso do Sul, Cidade Universitária 79070-900 Campo Grande, MS, Brazil; ^3^Federal University of Pernambuco, Av. Professor Moraes Rêgo, Recife, PE 50670-901, Brazil; ^4^Laboratório de Reatividade Cardiovascular, Federal University of Alagoas, Av. Lourival Melo Mota, Maceió, AL 57072-900, Brazil; ^5^Mountain Research Center (CIMO), Polytechnic Institute of Bragança, Campus Santa Apolónia, 5300-253 Bragança, Portugal

## Abstract

Obesity is an epidemic disease worldwide, associated with oxidative stress and the development of several other diseases. *Bauhinia rufa* (Bong.) Steud. is a native Brazilian Cerrado medicinal plant popularly used for the treatment of obesity. In this context, we investigated the chemical composition of the methanolic extract of *B. rufa* leaves (MEBr) and evaluated the antioxidant activity and its impact on the prevention and treatment of obesity in mice fed a high-fat diet (HFD 60%). Additionally, the acute oral toxicity of MEBr was evaluated. In MEBr, 17 glycosylated compounds were identified, including myricetin, quercetin, kaempferol, coumaroyl, cyanoglucoside, and megastigmane. *In vitro*, MEBr showed antioxidant activity in different methods: DPPH^•^, ABTS^•+^, FRAP, iron-reducing power, inhibition of *β*-carotene bleaching, and inhibition of DNA fragmentation. In human erythrocytes, MEBr increased the activities of antioxidant enzymes, superoxide dismutase, and catalase. Under oxidative stress, MEBr reduced oxidative hemolysis, and the malondialdehyde (MDA) levels generated in erythrocytes. Mice treated acutely with MEBr (2000 mg/kg) showed no signs of toxicity. During 90 days, the mice received water or MEBr simultaneously with HFD for induction of obesity. At this stage, MEBr was able to reduce the gain of subcutaneous white adipose tissue (WAT) and prevent the increase of MDA in the heart and brain. After 180 days of HFD for obesity induction, mice that received MEBr simultaneously with HFD (HFD-MEBr) in the last 60 days of treatment (120-180 days) showed a reduction of retroperitoneal and mesenteric WAT deposits and MDA levels in the heart, liver, kidney, and brain, compared to the HFD-Control group. These effects of MEBr were similar to mice treated with sibutramine (HFD-Sibutramine, 2 mg/kg). Combined, the results show that compounds from the leaves of *B. rufa* affect controlling oxidative stress and actions in the prevention and treatment of obesity. Thus, associated oxidative stress reduction and body composition modulation, in obese people, can contribute to the prevention of obesity-related comorbidities and improve quality of life.

## 1. Introduction

Obesity is a chronic inflammatory disease characterized by hypertrophy of adipocytes in adipose tissue, whose prevalence has doubled since 1980, becoming a public health problem worldwide [1]. Obese adipose tissue produces increased levels of reactive oxygen species (ROS). This increased production of ROS, concomitant with a neutralization imbalance of the endogenous antioxidant defense system, generates oxidative stress, a deleterious metabolic condition that affects macromolecules like DNA, lipids, and cellular proteins [2].

ROS from obese WAT can reach other organs, generating cell damage that leads to the development of obesity-associated diseases, such as cardiovascular, nonalcoholic steatohepatitis (NASH), kidney failure, and even cancer [2]. Furthermore, in 2019, with the COVID-19 pandemic, obese individuals also were at greater risk of severe respiratory cases when affected by COVID-19, raising the mortality rate [3].

From this perspective, due to the severity of obesity, drug and surgical treatments are indicated for patients to control obesity and related diseases [4]. However, the reduced number of antiobesity drugs, as well as the adverse effects promoted by synthetic drugs, and the high surgical risks call for the search for new therapeutic products [5].

Therefore, alternatives such as the use of therapeutic products, based on medicinal plants, have been the target of several researches for the treatment of diseases such as obesity. Studies of the chemical composition of species of the genus *Bauhinia* have revealed the presence of several bioactive substances. Among them, phenolic compounds, such as flavonoids, which have antioxidant activity and modulate lipolytic and lipogenic genes [6–9]. *Bauhinia rufa* (Bong.) Steud. (Fabaceae), popularly known as pata-de-vaca, unha-de-vaca, or catinga-de-tamanduá, is a native plant species of the Brazilian Cerrado used in traditional medicine as astringent, diuretic, anti-hyperlipidemic, antidiabetic, anorectic, and antiobesity [10]. Thus, considering the indications of use in traditional Brazilian medicine, as well as the presence of bioactive compounds described in other species of the genus, our hypothesis is that *Bauhinia rufa* will reduce fat mass and oxidative stress. However, studies that reveal its chemical profile and antiobesity activity are not available. In this context, we investigated the chemical composition of the methanolic extract of *B. rufa* (MEBr) leaves, its antioxidant activity, toxicity, and its preventive and therapeutic effects on obesity induced by a 60% high-fat diet (HFD).

## 2. Material and Methods

### 2.1. Vegetal Material and Extract Preparation

*B. rufa* leaves were collected (under authorization from the Biodiversity Authorization and Information System—SISBIO no. 45365-1) in Dourados, Mato Grosso do Sul, Brazil, with a localization of 22° 13′ 16” W 54° 48′ 2”. A voucher specimen (exsiccate), identified by a specialist, was deposited in the herbarium of the Federal University of Grande Dourados, Brazil (DDMS no. 4878).

The dry powdered vegetal material (360.60 g) was exhaustively macerated in methanol P.A. (5 l) for 30 days. The filtrate was concentrated under vacuum in a rotary evaporator at 45°C and thereafter lyophilized to obtain the methanolic extract of *B. rufa* leaves (MEBr). The MEBr yield was 16%.

### 2.2. Chemical Constituents

Ascorbic acid was determined according to the method of Barros et al. [11] and described by Rocha et al. [12]. The method is based on the extraction of ascorbic acid from MEBr (100 mg) using 1% metaphosphoric acid. The absorbance was measured within 30 min at 515 nm. The content of ascorbic acid was calculated based on the calibration curve of ascorbic acid (0.020-0.12 mg/ml). Results were expressed as mg of ascorbic acid/g of MEBr. The assays were carried out in triplicate.

Phenolic compounds were determined according to the method of Pinela et al. [13] and described by Rocha et al. [12]. The method was based on the extraction of phenolic compounds from MEBr (0.625 mg/ml) using HCl. The absorbance (A) at 280 nm was used to estimate total phenolic content, and gallic acid was used to calculate the standard curve (50-500 *μ*g/ml), and the results were expressed as mg of gallic acid equivalent (GAE) per gram of MEBr. A320 nm was used to estimate phenolic acids, and caffeic acid was used to calculate the standard curve (50-500 *μ*g/ml), and the results were expressed as mg of caffeic acid equivalent (CAE) per gram of MEBr. A360 nm was used to estimate flavonoids, and quercetin was used to calculate the standard curve (30-300 *μ*g/ml), and the results were expressed as mg of quercetin equivalent (QE) per gram of MEBr. All assays were carried out in triplicate.

### 2.3. HPLC-DAD-ESI-QTOF-MS/MS

MEBr (6 *μ*g) was injected into the Shimadzu LC-20AD UFLC chromatograph, coupled in line with a DAD and ESI-QTOF micrOTOF III (Bruker Daltonics). The DAD was monitored between 240 and 800 nm and MS between m/z 120 and 1200 in negative and positive modes. The MS/MS collision energy (CID) ranges from 45 to 65 eV. The stationary phase was a Kinetex C18 chromatography column (Phenomenex, 2.6 *μ*, 150 × 2.1 mm) and a gradient elution of water (Phase A) and acetonitrile (Phase B), both with 0.1% formic acid: 0-2 min. 3% of B; 2-25 min. 3-25% of B; and 25-35 min. 25-62% of B, followed by column washing and reconditioning (11 minutes). Flow rate was 0.3 ml/min. Data were processed using Data Analysis software version 4.2 (Bruker), and phenolic compounds were identified based on UV spectra, retention time, and fragmentation profile in comparison with the literature.

### 2.4. *In Vitro* Antioxidant Activities

The 2,2-diphenyl-1-picrylhydrazyl (DPPH^•^) radical scavenging activity was evaluated according to the method described by Campos et al. [14] and described by Rocha et al. [12]. Briefly, a 0.11 mM DPPH solution was mixed with different concentrations of MEBr (0.1 to 1000 *μ*g/ml). The absorbance was measured within 30 min at 517 nm. Ascorbic acid and butylated hydroxytoluene (BHT) were used as reference antioxidants. Three independent experiments were performed in triplicate.

The 2,2′-azino-bis(3-ethylbenzothiazoline-6-sulphonic acid) (ABTS^•+^) radical scavenging activity was evaluated according to the method described by Campos et al. [15] and described by Rocha et al. [12]. Briefly, ABTS solution (5 ml of 7 mM ABTS and 88 *μ*l of 140 mM potassium persulfate) was mixed with different concentrations of MEBr (0.1 to 1000 *μ*g/ml). The absorbance was measured within 6 min at 734 nm. Ascorbic acid and BHT were used as positive controls. Three independent experiments were performed in triplicate.

The ferric reducing antioxidant power (FRAP) assay was evaluated according to the method described by Pulido et al. [16] and described by Rocha et al. [12]. Briefly, FRAP reagent (10 ml of a 10 mmol/l 2,4,6-tris(2-pyridyl)-S-triazine (TPTZ) solution in 40 mmol/l HCl, 10 ml of 20 mmol/l ferric chloride hexahydrate (FeCl_3_·6H_2_O), and 100 ml of 0.3 mmol/l acetate buffer, pH 3.6) was mixed with different concentrations of MEBr (0.1 to 1000 *μ*g/ml). The absorbance was measured within 20 min at 595 nm. Ascorbic acid was used as a reference antioxidant. Three independent experiments were performed in triplicate.

The reducing power was evaluated according to the method described by Berker et al. [17] and described by Rocha et al. [12]. Briefly, a solution of 0.2 M phosphate buffer (pH 6.6) and 2.5 ml of potassium ferricyanide was mixed with different concentrations of MEBr (0.1 to 1000 *μ*g/ml) and incubated at 50°C for 20 min. Subsequently, 10% trichloroacetic acid (TCA) was added. An aliquot of 2.5 ml was withdrawn, and 2.5 ml of water followed by 0.5 ml of FeCl_3_·6H_2_O solution (0.1%) was added so that the final volume was 5.5 ml. The absorbance was measured within 2 min at 700 nm. Ascorbic acid was used as a reference antioxidant. Three independent experiments were performed in triplicate.

The *β*-carotene bleaching inhibition was evaluated according to the method described by Taga et al. [18] and described by Rocha et al. [12]. Briefly, an emulsion of *β*-carotene (1 ml of *β*-carotene, 20 mg linoleic acid, 200 mg Tween 40, and 50 ml of oxygenated distilled water) was mixed with different concentrations of MEBr (0.1 to 1000 *μ*g/ml). The absorbance was measured at 470 nm after 2 min at 50°C. Butylated hydroxyanisole (BHA) was used as a reference antioxidant. Three independent experiments were performed in triplicate.

### 2.5. DNA Fragmentation Induced by Hydrogen Peroxide

The assay of DNA fragmentation was performed according to Antunes et al. [19], with some modifications. A plasmidial DNA of 5,597 bp (4 *μ*l at 50 ng/*μ*l) was mixed with 4 *μ*l of MEBr (50-250 *μ*g/ml) and with 3% hydrogen peroxide. The samples were incubated in 302 nm transilluminator (UVT-312) at room temperature for 5 min. Then, the samples were subjected to electrophoresis on 2% agarose gel containing ethidium bromide (10 mg/ml). The gel was digitized using the Gel Doc™ EZ System (Bio-Rad) and analyzed using the Image Lab™ software. The average of 3 gels was used to determine the protection conferred by MEBr against DNA fragmentation.

### 2.6. Antioxidant Activities in Human Erythrocytes

The antioxidant assay in the model of human erythrocytes was evaluated according to the method described by Rocha et al. [20]. Human erythrocyte assays were performed after receiving approval from the Research Ethics Committee of the Federal University of Grande Dourados, MS, Brazil (process number 1.739.987 CEP).

#### 2.6.1. Antioxidant Enzyme Activity

As described by Rocha et al. [20]. Erythrocytes were incubated with different MEBr concentrations (50-250 *μ*g/ml) for 20 min at 37°C. The activity of antioxidant enzymes was normalized by hemoglobin (Hb). Superoxide dismutase (SOD) activity was determined using the Fluka® commercial kit (Sigma-Aldrich®, Seelze, Germany) according to the manufacturer's instructions, expressed as IU/Hb, mmol/l. Catalase (CAT) activity was spectrophotometrically determined by the hydrogen peroxide (H_2_O_2_) decomposition rate, according to the method described by Xu et al. [21], with modifications for microplates, expressed as *μ*mol/min/ml/mmol Hb. Glutathione peroxidase (GPx) activity was determined using the colorimetric method described by Paglia and Valentine [22], adapted for microplates, expressed as nmol/min/ml/Hb, nmol/l. Two independent experiments were performed in triplicate.

#### 2.6.2. Hemolysis, Oxidative Hemolysis, and Dosage of Malondialdehyde (MDA) in Erythrocytes

The antioxidant assay in the model of human erythrocytes was evaluated according to the method described by Rocha et al. [20]. Briefly, a 10% erythrocyte suspension was preincubated with different concentrations of MEBr (50-250 *μ*g/ml) for 30 min at 37°C. After 30 min, 2,2′-azobis (2-amidinopropane) dihydrochloride (AAPH) 50 mM was added. The absorbance was measured at 540 nm after 180 min at 37°C. For evaluation of MDA content, after the 180 min period, 10 nmol thiobarbituric acid (TBA) was added to the supernatant. The absorbance was measured at 532 nm after 45 min at 96°C. Ascorbic acid was used as a reference antioxidant. Three independent experiments were performed in triplicate.

### 2.7. *In Vivo* Studies

The experimental procedures with mice followed the rules of the National Council for Animal Experimentation Control (CONCEA) and were approved by the Ethics Committee for the Use of Animals of the Federal University of Grande Dourados (report no. 37/2015 CEUA/UFGD). Male C57Bl/6 mice weighing 20-30 g, adults, were obtained from the Central Vivarium of the Federal University of Grande Dourados, MS, Brazil. The mice were housed in microisolators (5 mice per microisolator), maintained in standard conditions (12 h of light and 12 h of dark, 22 ± 2°C), with access to water and feed *ad libitum*. Before the start of the experiment, the mice were acclimatized to the laboratory conditions. The dose for the *in vivo* tests was selected based on the acute toxicity study. The dose chosen was 10 times lower than the dose considered safe in this study.

#### 2.7.1. Acute Toxicity

The acute toxicity study was carried out according to protocols of the Organization for Economic Cooperation and Development (OECD) guidelines 425 [23].

#### 2.7.2. Diets

Standard diet AIN-93M (SD: carbohydrates 76%; protein 14% and lipids 10%) and high-fat diet HF9 60 (HFD 60%: carbohydrates 26%; protein 14% and lipids 60%) were purchased from PragSoluções Biociências (São Paulo, Brazil). SD possessed 377 kcal/100 g and HFD conferred a total caloric value of 576 kcal/100 g.

#### 2.7.3. Obesity Development

The effect of MEBr on the development of obesity was evaluated in HFD-fed mice in two phases: prevention and treatment.

1. Prevention of obesity development: mice received HFD simultaneously with water (HFD-Control) or MEBr, 200 mg/kg (HFD-MEBr) for 90 days. Standard control (SD-Control): mice received a standard diet and water for 90 days
2. Treatment of obesity: mice received HFD for 120 days, for induction of obesity. After this period, mice received HFD simultaneously with water (HFD-Control); sibutramine, 2 mg/kg (HFD-Sibutramine); or MEBr, 200 mg/kg (HFD-MEBr) for 60 days. Standard control (SD-Control): mice received a standard diet for 120 days; after this period, mice received water simultaneously, for 60 days

Body mass, water consumption, and feed were recorded weekly. MEBr, sibutramine, and water were administered using the intragastric gavage technique. At the end of the experimentation period, all mice were euthanized. The organs were removed and weighed and the levels of MDA were assessed [24]. The mice's blood was collected for biochemical analysis.

### 2.8. Statistical Analysis

The data obtained were expressed as mean ± standard error of the mean (SEM). For analysis and comparison between the experimental groups, a one-way analysis of variance (ANOVA) with Dunnett's post hoc test was used. All statistical analyzes were performed using the GraphPad Prism program, version 5.0. The data were considered significant when *P* < 0.05.

## 3. Results

### 3.1. Phytochemical Constituents

The concentrations of ascorbic acid, phenolic compounds, phenolic acids, and flavonoids determined in MEBr were 34 ± 2 *μ*g/g extract, 230 ± 14 mg GAE/g extract, 60 ± 4 mg CAE/g extract, and 92 ± 4 mg QE/g of extract, respectively.

### 3.2. Identification of Compounds

Seventeen compounds were identified in MEBr by HPLC-DAD-MS/MS (Figure S1, Supplementary Material) (Table 1).

Peak 1 (m/z 330.1188 [M+H]^+^, C_14_H_19_NO_8_) generated the fragment m/z 168 (C_8_H_10_NO_3_)^+^, referring to the loss of a glycoside group, and was putatively identified as cyanoglucoside with a hexose and a cyanoderivative with formula C_8_H_9_NO_3_; this class of compounds has been earlier reported in *Bauhinia* [25]. Compound 2 (m/z 327.1074 [M+H]^+^, C_15_H_18_O_8_) showed a UV spectrum compatible with a coumaroyl group and was identified according to data reported by Anttonen and Karjalainen [26] as coumaroyl-O-hexoside. Peaks 3 and 4 m/z 413 (C_19_H_34_O_8_) did not absorb in the monitored UV spectrum (240-800 nm) and according to data reported by Yoshikawa et al. [27], could be identified as megastigmane-O-hexoside.

The other compounds identified in the extract showed UV spectra compatible with a flavonol skeleton, with two bands centered at about 270-280 nm and 340-360 nm. This class was commonly found in the genus *Bauhinia* [6, 7]. Peaks 5, 6, 7, and 11 (m/z 379 [M-H]^−^, C_21_H_20_O_13_) showed the fragment m/z 316 [M-hexose]^−^ that violates the odd-electron rule and form a radical fragment, compatible with the loss of a hexoside group and were identified as myricetin-O-hexoside derivatives [28]. Compounds 8 and 10 (m/z 449 [M-H]^−^, C_20_H_18_O_12_) formed the same fragment of the aforementioned (m/z 316), however, in this case, referring to the loss of a pentoside, and were identified as myricetin-O-pentoside [7]. Compounds 12 and 13 m/z 463 [M-H]^−^, compatible with the molecular formula C_21_H_20_O_12_, had one oxygen less than myricetin-O-hexoside derivatives and generated the fragment m/z 300 [M-hexose]^−^ that violates the odd-electron rule and form a radical fragment, referring to the loss of a hexoside group, and being identified as quercetin-O-hexoside [29], as well as in compound 16 with m/z 447.0954 [M-H]^−^. In addition, a radical ion m/z 300 generated was identified as quercetin-O-deoxyhexoside [6]. Compound 15 (m/z 433.0754 [M-H]^−^, C_20_H_18_O_11_) was identified as quercetin-O-pentoside due to the loss of a pentoside group revealed by the m/z 300 fragment [M-pentose] [7], while compound 17 (m/z 315.0499 [M-H]^−^ C_16_H_12_O_7_) provided the m/z 315 [M-H]^−^ and was identified as methoxyquercetin, conferring with data reported by Farag et al. [6].

Finally, compound 14 (m/z 447.0908 [M-H]^−^, C_21_H_20_O_11_) presented an oxygen less than quercetin-O-hexoside and generated the radical fragment m/z 284.0299 [M-hexose]^−^, relative to the loss of a hexoside group, and was identified as kaempferol-O-hexoside [7].

All identified flavonoids have already been reported in the genus *Bauhinia* [6, 7, 29], but they are reported for the first time in the species of *B. rufa*.

### 3.3. MEBr *In Vitro* Antioxidants Activities

The antioxidant activity of MEBr was observed in the DPPH^•^, ABTS^•+^, FRAP, reducing power, and *β*-carotene bleaching assays (Table 2).

Compared to control, MEBr concentration capable of inhibiting 50% (IC_50_) of the DPPH^•^ and ABTS^•+^ radical was higher than ascorbic acid (approximately 3 times) and lower than BHT (approximately 3 times) (Table 2). The mean effective concentrations (EC_50_) of MEBr in the FRAP and reducing power tests were higher than ascorbic acid (approximately 4 and 5 times, respectively) (Table 2). In the *β*-carotene bleaching assay, MEBr showed an IC_50_ greater than BHA (approximately 52 times) (Table 2). The IC_50_ of ascorbic acid was not detected in the *β*-carotene bleaching assay (Table 2).

**Figure 1:**
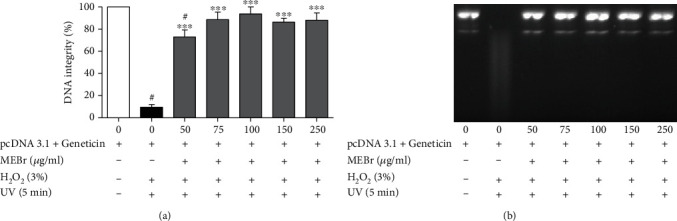
Effect of methanolic extract of *B. rufa* leaves (MEBr) on DNA fragmentation induced by hydrogen peroxide (H_2_O_2_) and ultraviolet (UV) light. (a) Percentage of DNA integrity and (b) representative image of plasmid DNA fragmentation ((a) pcDNA 3.1: plasmid cloning DNA). Values are expressed as mean ± SEM. ^#^*P* < 0.05 versus MEBr 0 *μ*g/ml; ^∗∗∗^*P* < 0.001 versus MEBr 0 *μ*g/ml+H_2_O_2_+UV.

MEBr prevented plasmid DNA fragmentation at all concentrations evaluated (50-250 *μ*g/ml) in a concentration-independent manner by approximately 84 ± 4%, when subjected to oxidative stress promoted by the combination of H_2_O_2_ and UV light (Figure 1).

**Figure 2:**
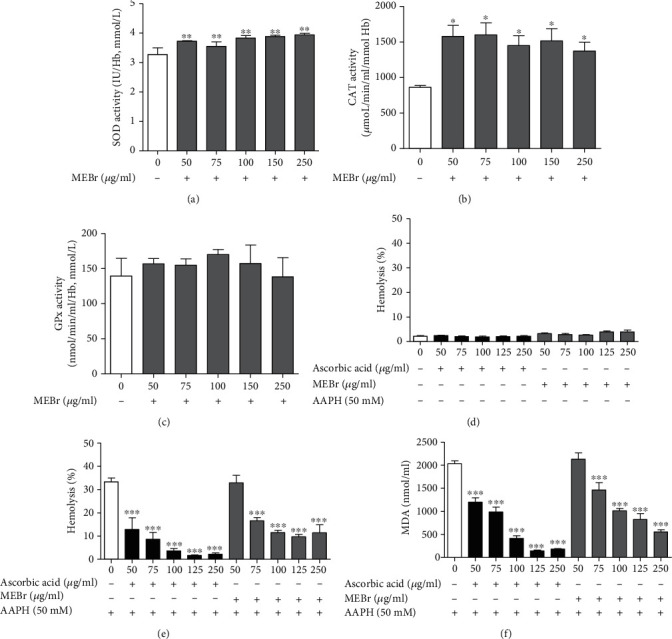
Effect of methanol extract of *B. rufa* leaves (MEBr) in human erythrocytes. (a) Superoxide dismutase (SOD), (b) catalase (CAT), and (c) glutathione peroxidase (GPx) enzymes, (d) hemolysis, (e) hemolysis induced by AAPH, and (f) MDA levels. AAPH: azobis(2-methylpropionamidine) dihydrochloride. Values are expressed as mean ± SEM. ^∗^*P* < 0.05; ^∗∗^*P* < 0.01; ^∗∗∗^*P* < 0.001 versus MEBr 0 *μ*g/ml (control).

The effect of MEBr on the activity of antioxidant enzymes SOD, CAT, and GPx, oxidative hemolysis, and MDA measurement in human erythrocytes is shown in Figures 2(a)–2(c). In human erythrocytes, MEBr increased the activity of the antioxidant enzymes SOD and CAT at all concentrations tested (50-250 *μ*g/ml) (Figures 2(a) and 2(b)) and did not alter the activity of the GPx enzyme (Figure 2(c)).

On one hand, incubation of human erythrocytes with MEBr alone did not promote hemolysis during 180 min of evaluation at the concentrations evaluated (Figure 2(d)), while on the other hand, MEBr reduces oxidative stress induced by the oxidizing agent AAPH in human erythrocytes (Figure 2(e) and 2(f)), compared to the control. At concentrations of 75-250 *μ*g/ml, MEBr inhibited hemolysis by 50 ± 4, 65 ± 3, 71 ± 3, and 65 ± 10%, respectively, compared to the control (Figure 2(e)). Furthermore, MEBr prevented MDA generation by 28 ± 6, 50 ± 2, 59 ± 5, and 73 ± 2% in a concentration-dependent manner (75, 100, 125, and 250 *μ*g/ml, respectively), compared to the control (Figure 2(f)).

### 3.4. MEBr *In Vivo* Activities

#### 3.4.1. Acute Toxicity

Table 3 shows the anthropometric and hematological parameters of mice treated with MEBr in the acute toxicity test. No mortality was observed during the experimental period after administration of 2000 and 5000 mg/kg MEBr. Mice treated at these doses of MEBr showed no changes in the anthropometric and hematological parameters evaluated, except for liver enlargement at the highest dose. Considering the data as a whole, the median lethal dose LD_50_ of MEBr was determined to be greater than 5000 mg/kg and the *in vivo* tests were done with a dose 10 times lower than the dose considered safe in this study (200 mg/kg).

#### 3.4.2. MEBr in the Prevention and Treatment of Obesity

The effect of MEBr on the development of obesity was evaluated in HFD-fed mice in two distinct phases: phase 1: prevention (90 days) and phase 2: treatment (120-180 days).

**Figure 3:**
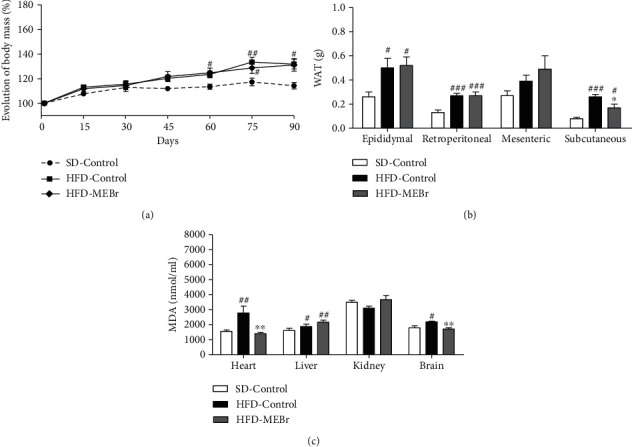
Effect of methanol extract of *B. rufa* leaves (MEBr) in C57Bl/6 mice. (a) Evolution of body mass, (b) white adipose tissue deposits, and (c) malondialdehyde (MDA) generation in organs in mice fed with HFD simultaneously with water (HFD-Control) or MEBr, 200 mg/kg (HFD-MEBr), for 90 days. Standard control (SD-Control): mice received a standard diet and water for 90 days. Values are expressed as mean ± SEM. *N* = 10 mice per group. ^#^*P* < 0.05 versus SD-Control; ^∗^*P* < 0.05 versus HFD-Control.

Mice fed for 90 days on HFD and water (HFD-Control) had an approximately twofold increase in body mass gain (Figure 3(a)) and a two- to threefold increase in WAT deposits (Figure 3(b)), compared to mice treated with a standard diet and water (SD-Control). Additionally, HFD increased serum triglycerides (Table 4) and MDA levels in the heart, liver, and brain by 1.2 to twofolds (Figure 3(c)), compared to mice in the SD-Control group. Together, these changes highlight the development of obesity in HFD-Control mice.

Regarding the altered parameters in this prevention phase (SD-Control versus HFD-Control), MEBr was able to prevent subcutaneous WAT gain by 35 ± 12% (Figure 3(b)) and inhibited the increase in MDA levels generated in the heart by 50 ± 3% and in the brain by 22 ± 4% (Figure 3(c)) in the HFD-MEBr group when compared to the HFD-Control group. HFD-MEBr mice showed increased calorie intake and water consumption, with no change in total body mass compared to the HFD-Control group (Table 4). In aggregate, these data highlight the beneficial effects of MEBr in preventing the development of obesity.

**Figure 4:**
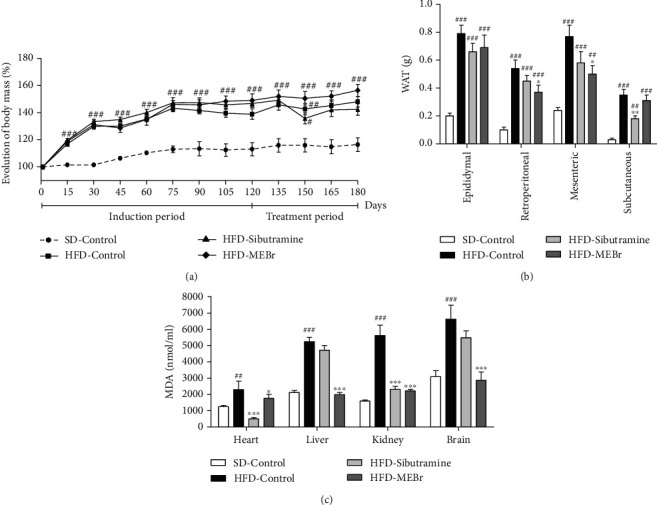
Effect of methanol extract of *B. rufa* leaves (MEBr) in C57Bl/6 mice. (a) Evolution of body mass, (b) white adipose tissue deposits, and (c) malondialdehyde (MDA) generation in organs in mice fed with HFD for 120 days to induce obesity. After this period, the mice received HFD concurrently with water (HFD-Control); sibutramine, 2 mg/kg (HFD-Sibutramine); or MEBr, 200 mg/kg (HFD-MEBr) for 60 days (120-180 days). Standard control (SD-Control): mice received a standard diet for 120 days; after this period, mice received water simultaneously, for 60 days (120-180 days). Values are expressed as mean ± SEM. *N* = 10 mice per group. ^#^*P* < 0.05 versus SD-Control; ^∗^*P* < 0.05 versus HFD-Control.

In the second phase of experimentation of obesity development (0-120 days) and simultaneous treatment with HFD (120-180 days), mice that received water (HFD-Control) showed approximately a threefold increase in body mass (Figure 4(a)) and deposits between 4 and 10 times (Figure 4(b)), compared to the SD-Control group. Combined, we find that HFD induced the development of obesity in the mice, including signs of oxidative stress in the different organs.

In obese mice, MEBr was able to reduce retroperitoneal WAT by 32 ± 10% and mesenteric WAT by 35 ± 8% compared to the HFD-Control group (Figure 4(b)). There was also a reduction in the generated MDA in all organs observed, with the reduction in the heart being 23 ± 11%, liver 62 ± 3%, kidney 60 ± 2%, and brain 57 ± 8% compared to the HFD-Control group (Figure 4(c)).

Regarding the anthropometric and biochemical parameters evaluated, there was no difference in the results observed between the treated HFD groups with sibutramine and MEBr, compared to HFD-Control, except for the reduction in blood glucose observed in the animals of the HFD-Sibutramine group compared to the HFD-Control (Table 5). These MEBr effects were similar to those observed in the sibutramine-treated mice, except for the reduction in glycemia (Table 5), and less noticeable effects in the reduction of the different WAT deposits, reducing only the subcutaneous one (Figure 4(b)), as well as in the absence of change in MDA levels generated in the liver and brain compared to HFD-Control (Figure 4(c)). Jointly, there are indications that MEBr has beneficial effects in the treatment of obesity, similar to sibutramine.

## 4. Discussion

This study shows for the first time the chemical composition and nontoxicity of *B. rufa* leaves and its beneficial pharmacological effects in stress oxidative control, prevention, and treatment of obesity in rodent experimental models.

Among the compounds present in MEBr are glycosylated flavonoids and ascorbic acid, which are described by their antioxidant activity, as already demonstrated for the flavonoids myricetin, quercetin, kaempferol, p-coumaroyl, megastigmane and cyanoglucoside [30–32]. The antioxidant activity of flavonoids is influenced especially by their chemical structure, particularly as a result of the number of double bonds and degree of hydroxylation [33]. The glycosylation of phenolic compounds improves their stability and bioactivity in biological systems [34], which may have intensified the antioxidant effects of MEBr. Mechanisms of direct capture of free radicals are conferred by the flavonoids hydroxyl groups [30–32, 35] and were observed in the tests performed with MEBr. Additionally, MEBr showed low iron reducing power, which may be beneficial because this ion catalyzes the production of reactive oxygen species that lead to lipid peroxidation and protein and DNA damage [36].

Collectively, they contributed to the protection by MEBr on DNA macromolecule against oxidative-induced damage. Flavonoids are relatively stable compounds because they can resist oxidation, high temperatures, and acidity variations [37]. This characteristic makes them interesting sources for product development because unneutralized ROS have been implicated in the development and progression of different diseases [35].

The ROS neutralization by antioxidant enzymes also contributes to the reduction of cell damage. MEBr increased the activity of the antioxidant enzymes SOD and CAT. It has been shown that components present in MEBr such as quercetin, myricetin, kaempferol, and p-coumaroyl increase the activity of the antioxidant enzymes SOD and CAT [38–43]. SOD is responsible for dismuting the radical superoxide anion (O2^•-^) to H_2_O_2_, which is neutralized by CAT, limiting the production of hydroxyl radicals (^•^OH) [44]. This is a beneficial effect since ^•^OH can attack directly cell membrane lipids, generating a cascade of lipid peroxidation with consequent cell damage. MEBr was able to reduce membrane peroxidation of erythrocytes in a state of oxidative stress, as demonstrated by the reduced levels of MDA generated. This cellular result probably evidences the effect of oxidative protection mechanisms observed previously in this study.

In addition to antioxidant activity, the substances present in MEBr have been indicated as regulators of lipid metabolism. Ascorbic acid has a lipolytic action in adipocytes [45]. The flavonoids quercetin, myricetin, and kaempferol downregulate lipogenic genes (C/EBP*α*, C/EBP*β*, PPAR*γ*, and SREBP-1c) and upregulate lipolytic genes (ATGL and HSL) [8, 9, 46, 47], resulting in reduced triglyceride accumulation in WAT adipocytes as observed for animals treated with MEBr.

The control of oxidative stress and WAT accumulation is fundamental for the prevention and control of obesity. The redox imbalance in biological systems can trigger or aggravate a pathological condition, such as obesity [35, 48]. ROS from obese WAT can reach other organs, promoting the development of other obesity-associated diseases [48]. Metabolic oxidative stress induces NASH progression [49], renal insufficiency [50], and cardiovascular diseases [51] in obesity.

After verifying the absence of mortality and signs of toxicity in mice acutely treated with high doses of MEBr, we investigated the effects of MEBr on the development of obesity. During the first phase of the experimentation, MEBr induced body mass remodeling, with a reduction in subcutaneous WAT, since there was no change in total body mass between the HFD-treated groups. In addition, MEBr increased caloric intake and water intake, suggesting an increase in metabolic rate. These data suggest that MEBr downregulates the process of lipogenesis.

This remodeling of body mass by MEBr has a positive impact on the prevention of obesity-associated diseases, as it prevents the oxidative state of the heart and brain, as observed by the reduced levels of MDA generated in mice treated with MEBr. These actions of MEBr indicate the beneficial effect in preventing the deleterious effects of obesity in the developmental stage of the disease.

In the second phase of experimentation, with obesity already installed, it was verified that MEBr reduced retroperitoneal and mesenteric WAT deposits, suggesting that the lipolytic action is combined with the antilipogenic action observed in the prevention phase. This result was accompanied by a reduction of MDA generated in the heart, liver, kidneys, and brain, indicating the beneficial effects of MEBr on the development of obesity-associated diseases, such as NASH, renal failure, and cardiovascular diseases. These effects of oxidative stress reduction in the liver and brain were not evident in the mice treated with sibutramine, serotonin, and norepinephrine reuptake inhibitor approved for the management of obesity.

Unlike other organs, the action on the brain depends on the passage of substances through the blood-brain barrier, which has been observed for some flavonoids [52, 53]. The reduction of oxidative stress in the brain has positive impacts on obesity-related neurovascular diseases [54].

These preclinical data are important findings for future clinical studies directed at developing new therapeutic possibilities against obesity [55].

## 5. Conclusion

Together, the results demonstrate for the first time that compounds from the leaves of *B. rufa* show antioxidant activity and have a preventive and therapeutic effect on obesity by reducing WAT accumulation and decreasing MDA levels in organs. Opening the perspective that the modulation of fat body mass associated with oxidative stress reduction in obese individuals may contribute to preventing comorbidities and thereby improving the quality of life.

## Figures and Tables

**Table 1:** The main compounds present in the methanolic extract of *B. rufa* leaves (MEBr) identified by HPLC-DAD-MS/MS.

Peak	RT (min)	UV (*λ* max)	Molecular formula	[M-H]^−^ (m/z)	[M+H]^+^ (m/z)	MS/MS (m/z)	MS/MS (m/z)	Compound/class
1	3	258	C_14_H_19_NO_8_	—	330.1188	—	168.0686	Cyanoglucoside
2	13.2	280	C_15_H_18_O_8_	325.0919	327.1074	—	—	Coumaroyl-O-hexoside
3	20.4	—	C_19_H_34_O_8_	389.2131	391.2212	—	—	Megastigmane-O-hexoside
4	21	—	C_19_H_34_O_8_	389.2137	391.2091			Megastigmane-O-hexoside
5	21.4	270/350	C_21_H_20_O_13_	479.082	481.0986	316.0189	319.0474; 273.0419; 245.0471	Myricetin-O-hexoside
6	21.8	270/350	C_21_H_20_O_13_	479.0811	481.0992	316.0204	—	Myricetin-O-hexoside
7	22	270/350	C_21_H_20_O_13_	479.0772	481.0977	—	—	Myricetin-O-hexoside
8	22.3	270/350	C_20_H_18_O_12_	—	451.881		319.0459; 273.0394	Myricetin-O-pentoside
9	22.3	270/350	—	—	563.2158	—	—	Unknown
10	23.1	260/351	C_20_H_18_O_12_	449.0703	451.0887	316.0201; 287.0269; 271.0220	319.0470; 273.0368; 165.0159	Myricetin-O-pentoside
11	23.5	256/348	C_21_H_20_O_12_	463.0863	465.1026	316.0210; 271.0257; 178.9971	319.0433; 245.0471; 153.0141	Myricetin-O-hexoside
12	23.9	258/350	C_21_H_20_O_12_	463.0866	465.1039	300.0248; 271.0235	303.0611	Quercetin-O-hexoside
13	24.4	268/350	C_21_H_20_O_12_	463.0864	465.1047	300.0253;	303.0513	Quercetin-O-hexoside
14	25.6	265/340	C_21_H_20_O_11_	447.0908	449.1081	284.0299	287.0552	Kaempferol-O-hexoside
15	25.8	260/351	C_20_H_18_O_11_	433.0754	435.0921	300.0257; 271.0254; 255.0295; 178,9950	303.0476; 257.0433; 229.0515; 201.0507	Quercetin-O-pentoside
16	26.5	255/347	C_21_H_20_O_11_	447.0904	449.1097	300.0257; 271.0238; 178.9948	303.0515; 257.0440; 229.0501	Quercetin-O-deoxyhexoside
17	32.7	270/360	C_16_H_12_O_7_	315.0499	317.0657	300.0277; 271.0242; 255.0283	302.0423; 274.0486; 228.0422	Methoxyquercetin

RT: retention time; min: minute; UV: ultraviolet light; m/z: mass-to-charge ratio; MS/MS: mass spectrometry.

**Table 2:** Antioxidant activity of the methanol extract of *B. rufa* leaves (MEBr).

Sample	DPPH^•^	ABTS^•+^	FRAP	Reducing power	*β*-Carotene bleaching
	IC_50_ (*μ*g/ml)		EC_50_ (*μ*g/ml)		IC_50_ (*μ*g/ml)
Ascorbic acid	3.06 ± 0.09	2.30 ± 0.08	33.58 ± 0.80	42.97 ± 0.70	ND
Lipophilic antioxidant	21.49 ± 0.39	22.77 ± 2.18	—	—	3.80 ± 0.10
MEBr	8.23 ± 0.46	5.24 ± 0.37	125.88 ± 4.81	231.11 ± 2.87	196.99 ± 20.51

Lipophilic antioxidant: BHT for DPPH^•^ and ABTS^•+^ and BHA for bleaching *β*-carotene; —: not done; ND: not detected. The results are expressed as mean ± SEM.

**Table 3:** Effect of the methanol extract of *B. rufa* leaves (MEBr) on anthropometric and hematological parameters of C57Bl/6 mice in an acute toxicity test for 14 days.

Parameters	Control	MEBr	
		2000 mg/kg	5000 mg/kg
Anthropometrics			
ΔBW (%)	3.42 ± 0.95	1.48 ± 1.61	2.99 ± 1.89
Water consumption (ml/day)	5.10 ± 0.23	4.83 ± 0.43	4.53 ± 0.42
Feed consumption (g/day)	3.44 ± 0.16	3.41 ± 0.22	3.33 ± 0.21
Liver (g/100 g of BW)	3.74 ± 0.05	3.83 ± 0.04	4.02 ± 0.06^#^
Lungs (g/100 g of BW)	0.62 ± 0.02	0.63 ± 0.02	0.61 ± 0.01
Kidneys (g/100 g of BW)	1.02 ± 0.02	1.03 ± 0.02	1.04 ± 0.01
Heart (g/100 g of BW)	0.49 ± 0.03	0.56 ± 0.02	0.55 ± 0.02
Hematological			
Red blood cell count (mm^6^)	9.75 ± 0.16	9.65 ± 0.10	9.66 ± 0.08
Hemoglobin (g/l)	13.82 ± 018	13.74 ± 0.18	14.17 ± 0.17
MCV (fl)	55.92 ± 0.33	56.26 ± 0.28	56.87 ± 0.16
MCH (pg)	14.23 ± 0.06	14.24 ± 0.07	14.46 ± 0.21
MCHC (g/dl)	25.34 ± 0.23	25.32 ± 0.19	25.78 ± 0.14
Platelets (mm^3^)	904.60 ± 52.23	883.40 ± 26.46	941.33 ± 8.59
Leukocyte count (mm^3^)	3.58 ± 0.29	3.17 ± 0.36	3.13 ± 0.24
Neutrophils (%)	6.38 ± 2.30	4.14 ± 0.69	3.50 ± 0.60
Lymphocytes (%)	93.08 ± 2.31	95.30 ± 0.64	95.93 ± 0.83
Monocytes (%)	0.30 ± 0.16	0.36 ± 0.12	0.45 ± 0.26

ΔBW (%): variation in % between final and initial body weight; MCV: mean corpuscular volume; MCH: mean corpuscular hemoglobin; MCHC: mean corpuscular hemoglobin concentration. Values are expressed as mean ± SEM. *N* = 5 mice per group. ^#^*P* < 0.05 versus control.

**Table 4:** Effect of methanol extract of *B. rufa* leaves (MEBr) in anthropometric and biochemical parameters of C57Bl/6 mice fed with HFD simultaneously with water (HFD-Control) or MEBr, 200 mg/kg (HFD-MEBr) for 90 days. Standard control (SD-Control): mice received a standard diet and water for 90 days.

Parameters	SD-Control	HFD-Control	HFD-MEBr
Anthropometrics			
ΔBW (%): 0-90 days	14.33 ± 2.60	31.89 ± 3.96^#^	31.24 ± 5.05
Water intake (ml day)	2.89 ± 0.05	2.38 ± 0.02^###^	3.20 ± 0.02^∗∗∗^
Food intake (kcal/day)	11.31 ± 0.31	14.55 ± 0.25^###^	16.71 ± 0.20^∗∗∗^
Heart (g)	0.13 ± 0.01	0.12 ± 0.01	0.13 ± 0.01
Liver (g)	0.95 ± 0.03	0.90 ± 0.02	0.98 ± 0.04
Kidneys (g)	0.28 ± 0.01	0.25 ± 0.01	0.27 ± 0.01
Brain (g)	0.41 ± 0.01	0.41 ± 0.01	0.41 ± 0.01
Biochemistry			
Glycemia (mg/dl)	127.11 ± 9.38	109.90 ± 11.71	113.40 ± 9.03
Triglycerides (mg/dl)	178.22 ± 11.82	225.20 ± 7.11^##^	238.00 ± 12.32
Cholesterol (mg/dl)	165.11 ± 3.78	167.80 ± 1.58	165.00 ± 0.38

ΔBW (%): % variation between final and initial body weight. Values are expressed as mean ± SEM. *N* = 10 mice per group. ^#^*P* < 0.05 versus SD-Control; ^∗^*P* < 0.05 versus HFD-Control.

**Table 5:** Effect of methanol extract of *B. rufa* leaves (MEBr) in anthropometric and biochemical parameters of C57Bl/6 mice fed with HFD for 120 days to induce obesity. After this period, the mice received HFD concurrently with water (HFD-Control); sibutramine, 2 mg/kg (HFD-Sibutramine); or MEBr, 200 mg/kg (HFD-MEBr) for 60 days (120-180 days). Standard control (SD-Control): mice received a standard diet for 120 days; after this period, mice received water simultaneously, for 60 days (120-180 days).

Parameters	SD-Control	HFD-Control	HFD-Sibutramine	HFD-MEBr
Anthropometrics				
ΔBW (%): 0-180 days	15.41 ± 4.83	47.04 ± 5.01^###^	43.09 ± 4.80	56.48 ± 4.22
ΔBW (%): 120-180 days	−0.05 ± 0.76	1.73 ± 1.61	−2.64 ± 1.72	−3.05 ± 1.87
Water intake (ml day)	4.19 ± 0.13	2.56 ± 0.09^###^	2.56 ± 0.06	2.67 ± 0.05
Food intake (kcal/day)	14.00 ± 0.42	15.79 ± 0.46^#^	16.29 ± 0.39	15.91 ± 0.27
Heart (g)	0.15 ± 0.01	0.14 ± 0.01	0.15 ± 0.01	0.14 ± 0.01
Liver (g)	1.18 ± 0.05	1.24 ± 0.05	1.33 ± 0.05	1.08 ± 0.04
Kidneys (g)	0.33 ± 0.02	0.31 ± 0.01	0.34 ± 0.01	0.29 ± 0.01
Brain (g)	0.44 ± 0.01	0.42 ± 0.01	0.42 ± 0.01	0.45 ± 0.01
Biochemistry				
Glycemia (mg/dl)	84.91 ± 3.38	138.13 ± 6.90^###^	107.38 ± 8.31^∗^	135.44 ± 10.42
Triglycerides (mg/dl)	199.36 ± 13.93	202.63 ± 15.17	175.00 ± 11.31	180.11 ± 14.41
Cholesterol (mg/dl)	161.64 ± 1.61	200.50 ± 13.79^##^	186.50 ± 6.87	175.63 ± 3.71

ΔBW (%): % variation between final and initial body weight. Values are expressed as mean ± SEM. *N* = 10 mice per group. ^#^*P* < 0.05 versus SD-Control; ^∗^*P* < 0.05 versus HFD-Control.

## Data Availability

All the results are included in the submitted manuscript and supplemental file.

## Supplementary Materials

Supplementary Materials Base peak chromatogram figure in negative ionization mode of methanolic extract of *B. rufa* leaves (MEBr). Peaks 1-17.
