# Supplementary material for: Beneficial Effects of Bauhinia rufa Leaves on Oxidative Stress, Prevention, and Treatment of Obesity in High-Fat Diet-Fed C57BL/6 Mice
Source: Oxid Med Cell Longev. 2022 Nov 25;2022:8790810. doi: 10.1155/2022/8790810 (PMC9718629; doi:10.1155/2022/8790810)
Supplement: Supplementary Materials — Base peak chromatogram figure in negative ionization mode of methanolic extract of B. rufa leaves (MEBr). Peaks 1-17. [file 8790810.f1.pdf]

Supplemental file

Research Article: Beneficial effects of *Bauhinia rufa* leaves on oxidative stress, prevention and treatment of obesity in high-fat diet-fed C57BL/6 mice

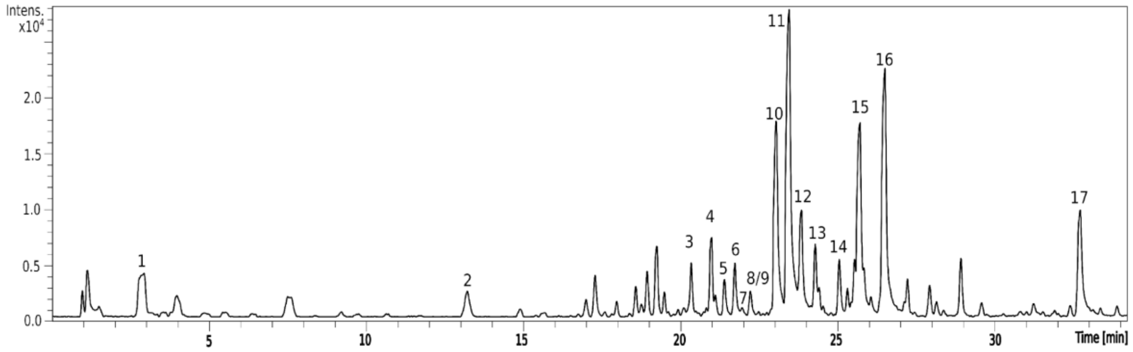

Fig. S1. Base Peak chromatogram in negative ionization mode of methanolic extract of *B. rufa* leaves (MEBr). Peaks 1-17 were identified in Table 1.
